# Divergence time of mites of the family Laelapidae based on mitochondrial barcoding region

**DOI:** 10.1371/journal.pone.0279598

**Published:** 2023-02-14

**Authors:** Huijuan Yang, Ting Chen, Wenge Dong

**Affiliations:** Institute of Pathogens and Vectors, Yunnan Provincial Key Laboratory for Zoonosis Control and Prevention, Dali University, Dali, Dali Bai Autonomous Prefecture, Yunnan, China; Shiv Nadar University, INDIA

## Abstract

Using the mitochondrial barcoding region to correlate research with 58 species in 19 genera of the family Laelapidae with the aim of determining the origin, phylogenetic relationships, and biogeographic historical distribution characteristics of mites in the family Laelapidae. Phylogenetic trees were obtained using Bayesian inference (BI) and Maximum-likelihood (ML) methods, based on three fossil records calibrated as molecular clock nodes, to estimate the divergence time of mites in the family Laelapidae as well as to apply Dispersal-Extinction-Cladogenesis (DEC) analyses to obtain biogeographic history inferences. The result showed species of the genera *Hyperlaelaps* and *Haemolaelaps* and some species of the genus *Androlaelaps* in the family Laelapidae were divided into clades of the genus *Laelaps* in both the BI and ML trees. Divergence time estimates and biogeographic history analysis revealed that the family Laelapidae likely diverged from other taxa during the Middle Jurassic (ca. 156.73 Mya), with Asia considered the most likely ancestral region for the family Laelapidae. Species of various genera began to undergo massive diversification events during the Cenozoic Tertiary. The results suggest that some genera in the family Laelapidae need to be re-defined or new genera need to be established; the Late Cretaceous to Late Neogene warm period would have promoted the divergence and expansion of species in the family Laelapidae. The divergence and dispersal of the family Laelapidae species is most likely a joint response to the continued northward drift of the Indian plate away from the Gondwana paleo-continent and gradually closer to Asia during the Late Cretaceous and the geological activity of the Tibetan Plateau during the Cenozoic Tertiary. The results strengthen our understanding of the origin and evolution of species in the family Laelapidae.

## Introduction

Laelapidae belongs to Animalia, Arthropoda, Arachnida, Acari, Parasitiformes, Gamasina, Dermanyssoidea [1, 2]. The family Laelapidae describes a large number of species, consisting of about 90 genera and more than 1300 species [3]. The general ecological habits and basic life history processes of mites of the family Laelapidae are five stages: egg, larvae, first nymph, second nymph and adults [4]. The mites of the family Laelapidae are often exclusively or facultatively parasitic on the body surfaces of mammals (especially rodents), birds, and reptiles. Specialized parasitic mites permanently parasitize the bodies of rodents, while facultative mites are found in the nests of rodents or in the rodents themselves (some of which feed on organic matter and other small arthropods) [5]. Turk (1945) and Allred (1969) described *Eulaelaps stabularis* as the most common mite found in the nests and bodies of rodents and insectivores [6, 7]. The mites of the family Laelapidae are closely associated with medicine and belong to the category of medical gamasina mites. The mites of the family Laelapidae found on the bodies of small rodents are generally considered to be a medically important group of arthropods, some of which are considered potential vectors of zoonoses diseases. For example, *Laelaps jettmari*, *Eulaelaps stabularis*, and *Haemolaelaps glasgowiare*, which carry the renal syndrome hemorrhagic fever virus (HFRS) [8–11]. There are also species of the family Laelapidae that are only distributed in certain areas, such as species of the genus *Tropilaelaps*, an ectoparasite of honeybees, which so far exists only in the Asian continent. There are also species that are important as predatory mites, such as *Stratiolaelaps scimitus*, which have been commercially produced and used in Europe and the United States to control mushrooms and greenhouse pests [12, 13].

The species of Dermanyssoidea lack consistent morphological characters, and the family Laelapidae is a large and important family in Dermanyssoidea. Species of the family Laelapidae differ greatly from other families of mites in Dermanyssoidea by their external morphology [14]. Because of the diversity of Laelapidae species and their importance to human beings, a large number of Laelapidae species have been thoroughly studied in morphological classification, distribution and new species reports [15]. The mites of the family Laelapidae have a small body size, which is a considerable obstacle to the development of molecular techniques, and the evolution of many Laelapidae family species seems to have proceeded by mimetic parasitism, which makes phylogenetic classification difficult because highly derived parasitic taxa often have the same characteristics as ancestral taxa, not because of relatedness, but because they have secondary loss characteristics [16]. Therefore, at taxonomic level studies, the phylogenetic relationships of species in the family Laelapidae have been controversial to the extent that they have not been well resolved. Casanueva (1993) made the first attempt to assess the internal relationships of the family Laelapidae using phylogenetic systematics by analyzing 83 morphological characters from 50 genera of the family Laelapidae and found that the family Laelapidae is a monophyletic group [17]. Nevertheless, because most species in the family Laelapidae have similar appearance and lack valid homologous morphological characters, traditional classification systems are challenged and phylogenetic relationships based on morphological classification may not be clear. One example is Strong (1995), based on Casanueva (1993), who reconstructed the phylogenetic relationship of the family Laelapidae by analyzing 55 taxa and 153 morphological characters of the family Laelapidae. The results showed that the phylogenetic relationships of the genus *Hypoaspis* in the family Laelapidae were unclear, resulting in the inability to confirm the monophyly or polyphyly of the family Laelapidae [16]. Since the 21st century, DNA barcoding technology has rapidly developed and gradually become one of the main methods for phylogenetic studies of mites [18]. On this basis, Dowling and O’Connor (2010) performed a phylogenetic reconstruction of Dermanyssoidea species using 18S and 28S genes, and the results showed that the family Laelapidae is a polyphyletic group [19]. In contrast, Li et al (2019) performed a phylogenetic reconstruction of Mesostigmata species using the mitochondrial (mt) genome, the results showed that the family Laelapidae was reverted back to monophyletic [20].

Mites have a long evolutionary history dating back to at least 410 million years ago (Mya) [21]. The evolutionary history of mites is poorly understood by most scholars and, as a result, they remain one of the least studied major branches of the animal tree of life [22]. The fossil record does not reflect the diversity of mites in the family Laelapidae, and no fossil record of the family Laelapidae mites has been found to date. As suggested by Dunlop et al. (2003 and 2014), one possible reason for this situation is that the vast majority of species in the family Laelapidae are parasitic on the surface of rodent bodies or in nests, and few species inhabit the bark of trees, which would then be less likely to be preserved as inclusions in amber; Another reason may be that the mites in the family Laelapidae are small and largely lack hard exoskeletons, making them difficult to fossilize or to distinguish [23, 24]. Only Dunlop and Selden’s team (2009) recorded a partial fossil record of the Acari taxon [25].

Molecular biogeographic history studies provide important insights into species range variation [26, 27]. A phylogeographic approach allows an in-depth exploration of the extent of species diversity and also allows observation of the historical processes that have contributed to the current geographic distribution of individuals [28, 29]. Given the rich diversity of gamasina mite species and the variety of geographic distribution types, species divergence, and subspecies formation in the family Laelapidae, there are few studies on the origin of species in the family Laelapidae and the time of divergence from other mites, and no studies have been conducted on the biogeographic history of the family Laelapidae. Thus, in this study, we determined the *cox1* gene sequences of five species of the family Laelapidae, combined with the *cox1* gene sequences of some species of the family Laelapidae downloaded from the GenBank database, and used a combination of molecular phylogeny and divergent chronological assessment to reconstruct the phylogenetic relationships of mites in the family Laelapidae, and on this basis, we further explored the divergence time and biogeographic history within the family Laelapidae species. The aim was to better reveal the genetic background and causes of divergence of species in the family Laelapidae at the molecular level, and to provide a framework for further studies on the evolution and divergence of species in the family Laelapidae in the future. Sequence information for the species of the family Laelapidae used for analysis in this study is presented in Table 1.

**Table 1:** The mitochondrial *cox1* gene of the family Laelapidae used in this study.

Family	Geneus	Species	Accession no.	Length	AT%	Region
Laelapidae	*Tropilaelaps*	*Tropilaelaps clareae*	EF025458	538	68.8	A
		*Tropilaelaps koenigerum*	EF025449	538	67.5	A
		*Tropilaelaps mercedesae*	KY865195	672	65.9	A
		*Tropilaelaps thaii*	EF025452	538	67.3	A
	*Stratiolaelaps*	*Stratiolaelaps lamington*	AY184369	1025	70.5	E
		*Stratiolaelaps lorna*	AY184366	1025	70.7	E
		*Stratiolaelaps marilyn*	AY184365	1299	73.7	E
		*Stratiolaelaps scimitus*	AY184367	1025	69.9	E
	*Pogonolaelaps*	*Pogonolaelaps canestrinii*	OL863238	1179	70.6	B
	*Pneumolaelaps*	*Pneumolaelaps fuscicolens*	MW367916	1188	66.7	A
	*Laelaspis*	*Laelaspis astronomicus*	MW367908	620	72.7	A
	*Laelaps*	*Laelaps fukienensis*	OL806574	438	72.4	A
		*Laelaps chini*	OL806586	438	71.5	A
		*Laelaps schatzi*	MK716211	620	72.7	B
		*Laelaps agilis*	MZ048462	582	74.6	B
		*Laelaps echidninus*	OL780835	421	73.2	A
		*Laelaps nuttalli*	OL810027	418	73.2	A
		*Laelaps muricola*	KU166786	644	73.0	D
		*Laelaps giganteus*	KU166639	644	71.4	D
		*Laelaps kochi*	MG414008	653	71.5	C
		*Laelaps taingueni*	KF437542	658	71.0	C
		*Laelaps hilaris*	MZ048456	582	72.0	B
		*Laelaps liui*	OM992254 (This study)	437	68.9	A
		*Laelaps turkestanicus*	OM992256 (This study)	438	70.5	A
		*Laelaps algericus*	OM992257 (This study)	400	73.8	A
		*Laelaps clethrionomydis*	OM754649	1026	71.6	A
	*Hypoaspis*	*Hypoaspis aculeifer*	KF966617	658	70.8	A
		*Hypoaspis miles*	FM210173	445	70.8	B
		*Hypoaspis linteyini*	MK270530	1539	68.4	A
		*Hypoaspis pavlovskii*	OM992258 (This study)	428	69.1	A
		*Hypoaspis digitalis*	OM992259 (This study)	429	60.6	A
		*Hypoaspis pentodoni*	OL863237	1179	66.0	B
	*Gaeolaelaps*	*Gaeolaelaps debilis*	MW367907	822	70.9	A
		*Gaeolaelaps minor*	OL863235	1179	71.2	B
		*Gaeolaelaps nolli*	MW367912	813	67.5	A
		*Gaeolaelaps aculeifer*	MH983647	658	66.9	A
	*Hyperlaelaps*	*Hyperlaelaps microti*	MZ048468	582	72.3	B
	*Holostaspis*	*Holostaspis isotricha*	MW367904	1188	65.7	A
		*Holostaspis montana*	MW367905	1188	70.5	A
	*Haemolaelaps*	*Haemolaelaps traubi*	OL810029	440	71.8	A
	*Gymnolaelaps*	*Gymnolaelaps myrmecophilus*	MW367911	1188	65.8	A
	*Echinonyssus*	*Echinonyssus sp*.	MN354695	639	70.4	C
		*Echinonyssus isabellinus*	MG408438	657	64.8	C
	*Cosmolaelaps*	*Cosmolaelaps cuneifer*	MW367917	855	66.8	A
		*Cosmolaelaps dendrophilus*	MW367903	1188	62.1	A
		*Cosmolaelaps lutegiensis*	OL863236	1179	72.9	B
		*Cosmolaelaps neocuneifer*	MW367913	1188	67.8	A
		*Cosmolaelaps rectangularis*	MW367910	1188	65.7	A
		*Cosmolaelaps vacua*	MW367906	822	62.4	A
	*Coleolaelaps*	*Coleolaelaps sp*.	AY184371	1001	72.5	E
		*Coleolaelaps cf*. *liui*	MK270524	1569	62.1	A
	*Androlaelaps*	*Androlaelaps marshalli*	KF805856	644	72.5	D
		*Androlaelaps casalis*	MH983844	657	71.0	B
	*Neocypholaelaps*	*Neocypholaelaps apicola*	KP966315	709	72.9	B
		*Neocypholaelaps indica*	MF040695	707	70.3	A
	*Myonyssus*	*Myonyssus gigas*	MZ048469	582	70.1	B
	*Haemogamasus*	*Haemogamasus nidi*	MZ049956	582	64.9	B
		*Haemogamasus ambulans*	MG414996	657	66.7	C

A: Asia; B: Europe; C: North American; D: Africa; E: Oceania

## Materials and methods

### Collection of mites

Mite specimens were collected from the body surfaces of 4 small mammal species (*Berylmys bowersi*, *Niviventer confucianus*, *Mus caroli*, and *Eothenomys miletus*) in Lijiang, Yunnan Province, China. Mite samples were either used immediately for DNA extraction or preserved in 95% ethanol at -20°C prior to DNA extraction. In addition, specimens of mite were also mounted to slides as vouchers, using Hoyer’s medium for morphological check with a Zeiss A2 (microphoto camera AxioCam MRc) microscope. The specimens and vouchers were deposited at the Institute of Pathogens and Vectors at Dali University in China. Small mammal capture protocols and procedures were approved by the animal ethics committees at Dali University. The approval ID is MECDU-201806-11.

### DNA extraction, mt genome amplification, and sequencing

Genomic DNA was extracted from individual mites with the DNeasy Blood and Tissue Kit (QIAGEN). One pair of cox1 gene primers (Sense: 5’-GGAGGATTTGGAAATTGATTAGTTCC-3’; Anti sense: 5’-CCCGGTAAAATTAAAATATAAACTTC-3’) [30, 31], were used to amplify fragments of the cox1 genes. PCR cycle conditions for the gene amplification were: 3 min at 94°C, over 40 cycles of 1 min at 94°C, 1 min at 52°C, 1 min at 72°C, and a final extension step of 10 min at 72°C. PCR products were analyzed by electrophoresis on a 1% agarose gel. The amplified gene fragments were sequenced directly using the double deoxy chain termination method at the ThermoFisher Scientific Genome Sequencing Facility (Guangzhou).

### Data analysis

Raw sequences were edited and assembled with SeqMan 7.1.0 [32] and aligned using the Muscle algorithm in MEGA X software [33]. The base composition of the sequences was analyzed using Geneious Prime 2021.1.1 [34]. Conserved sites, variant sites, and parsimony-informative sites of the sequences were calculated in MEGA X [33]. Next, the saturation analysis of sequence base substitutions was analyzed using DAMBE software [35]. The DnaSP 5.0 [36] software was used to calculate the ratio of non-synonymous (Ka) and synonymous (Ks) substitutions in the sequence.

### Phylogenetic analysis

Phylogenetic relationships of the family Laelapidae were constructed using Bayesian inference (BI) methods and Maximum-likelihood (ML) methods. PartitionFinder 2.1.1 [37] was used to determine the appropriate models for the first+ second, third codon positions for the construction of Bayesian trees for the *cox1* gene, and the best-fit models were found to be GTR+I+G, and GTR+G, respectively, according to the Bayesian Information Criterion (BIC). The best-fitting model for the Maximum-likelihood (ML) approach was found to be GTR + F + I + G4 using ModelFinder2 [38]. BI and ML analysis was performed in PhyloSuite [39]. For the Bayesian systematics analysis, a total of 20,000,000 generations were run, with sampling every 1000 generations and the first 25% of the trees burned and discarded to ensure sample independence, and four Monte Carlo Markov chains (MCMC) were run. Stationarity was considered to be reached when the average standard deviation of split frequencies was less than 0.01. To estimate the support of the Bayesian tree, we calculated the Bayesian posterior probability (PP). For the Maximum-likelihood systematics analysis, we compute the branching reliability (bootstrap probability, BP) with 50,000 ultra-fast bootstrap replications. The -bnni option was applied to minimize the risk of overestimating support values. The constructed phylogenetic tree was viewed and edited using FigTree 1.4.4 [40]. *Limulus polyphemus* and *Carcinoscorpius rotundicauda* (GenBank accession numbers: KT959421 and MF363154) were used as outgroups.

### Divergence time estimation

A divergence time tree for the family Laelapidae was constructed using BEAST 1.8.4 [41]. The prior category of the tree was set to Yule Process. 600 million generations of Markov chain Monte Carlo were run, sampled every 4000 generations. The fossil records of the families Ixodidae (100 Mya), Parasitidae (44–49 Mya) and Digamasellidae (>16 Mya) were used as calibration points [25, 42, 43]. The three time calibration points are embedded in the corresponding node locations. Run the subroutine BEAUti under BEAST 1.8.4 software, input the data file, and generate an xml file to import into BEAST. The TreeAnnotator 1.8.4 component of the BEAST package was used to discard the initial 10% of samples burned [44]. The convergence of the chain was confirmed using Tracer 1.7 [45] to ensure that the effective sample sizes (ESSs) of the parameters were greater than 200. The file generated by BEAST was opened using FigTree 1.4.4 [40] to obtain the divergence times for each clade. Sequence information for species of the families Ixodidae, Parasitidae, and Digamasellidae is presented in Table 2.

**Table 2:** Sequence information of species in the families Ixodidae, Parasitidae and Digamasellidae in this study.

Family	Genus	Species	Accession no.
Ixodidae	*Ixodes*	*Ixodes acuminatus*	MN308056
		*Ixodes collaris*	KR902756
		*Ixodes rubicundus*	GU437875
		*Ixodes ricinus*	MZ305543
		*Ixodes kaiseri*	MZ305531
		*Ixodes hexagonus*	MZ305530
		*Ixodes frontalis*	MZ305529
		*Ixodes scapularis*	MN348664
		*Ixodes marxi*	MN347960
		*Ixodes pacificus*	MN360338
		*Ixodes kingi*	MN359921
		*Ixodes muris*	MN358425
		*Ixodes soricis*	MN354578
		*Ixodes cookei*	MH338173
		*Ixodes granulatus*	MG721051
		*Ixodes spinipalpis*	MG414701
		*Ixodes pavlovskyi*	MG210488
		*Ixodes ovatus*	MH319670
		*Ixodes gregsoni*	KY370928
		*Ixodes affinis*	KX360422
Parasitidae	*Parasitus*	*Parasitus fimetorum*	MH983580
		*Parasitus wangdunqingi*	MK270528
		*Parasitus hyalinus*	MH983578
		*Parasitus loricatus*	MN906455
		*Parasitus beta*	MW004856
	*Poecilochirus*	*Poecilochirus carabi*	MW890886
		*Poecilochirus necrophori*	MW890935
		*Poecilochirus monospinosus*	MW890931
		*Poecilochirus subterraneus*	MW890956
		*Poecilochirus carabi sensu*	MW890816
		*Poecilochirus austroasiaticus*	MW890765
	*Pergamasus*	*Pergamasus brevicornis*	MW367970
		*Pergamasus vagabundus*	HM887562
		*Pergamasus mirabilis*	MG414271
		*Pergamasus misellus*	MG409917
		*Pergamasus crassipes*	MN347132
Digamasellidae	*Dendrolaelaps*	*Dendrolaelaps longiusculus*	MH983744
		*Dendrolaelaps reticulosus*	MG409996
		*Dendrolaelaps presepum*	MH983568
		*Dendrolaelaspis lobatus*	MH983801

### Infinite-sites plots

Infinite-sites plots were used to investigate the effect of sequence and calibration information on reducing the uncertainty (width of the posterior confidence interval) in the divergence time estimates [46]. The width of the confidence interval (CI) around the posterior age estimate is linearly related to the mean posterior age estimate as the molecular sequence data approaches infinity. We plot infinite-sites plots of the data in the same way and fit a straight line through the origin to the data.

### Biogeographic analysis

The geographic range of the family Laelapidae was divided into five regions: (A) Asia, (B) Europe, (C) North America, (D) Africa, and (E) Oceania. Model testing using BIOGEOBARES in Rasp software as a means of determining the best-fit model for reconstructing the possible biogeographic history of the family Laelapidae [47] to reconstruct the possible biogeographic history of the family Laelapidae. The maximum number of regions per node allowed in the ancestral distribution was 4. Other parameters were automatically optimized.

## Results

### Sequence analysis

**Fig 1:**
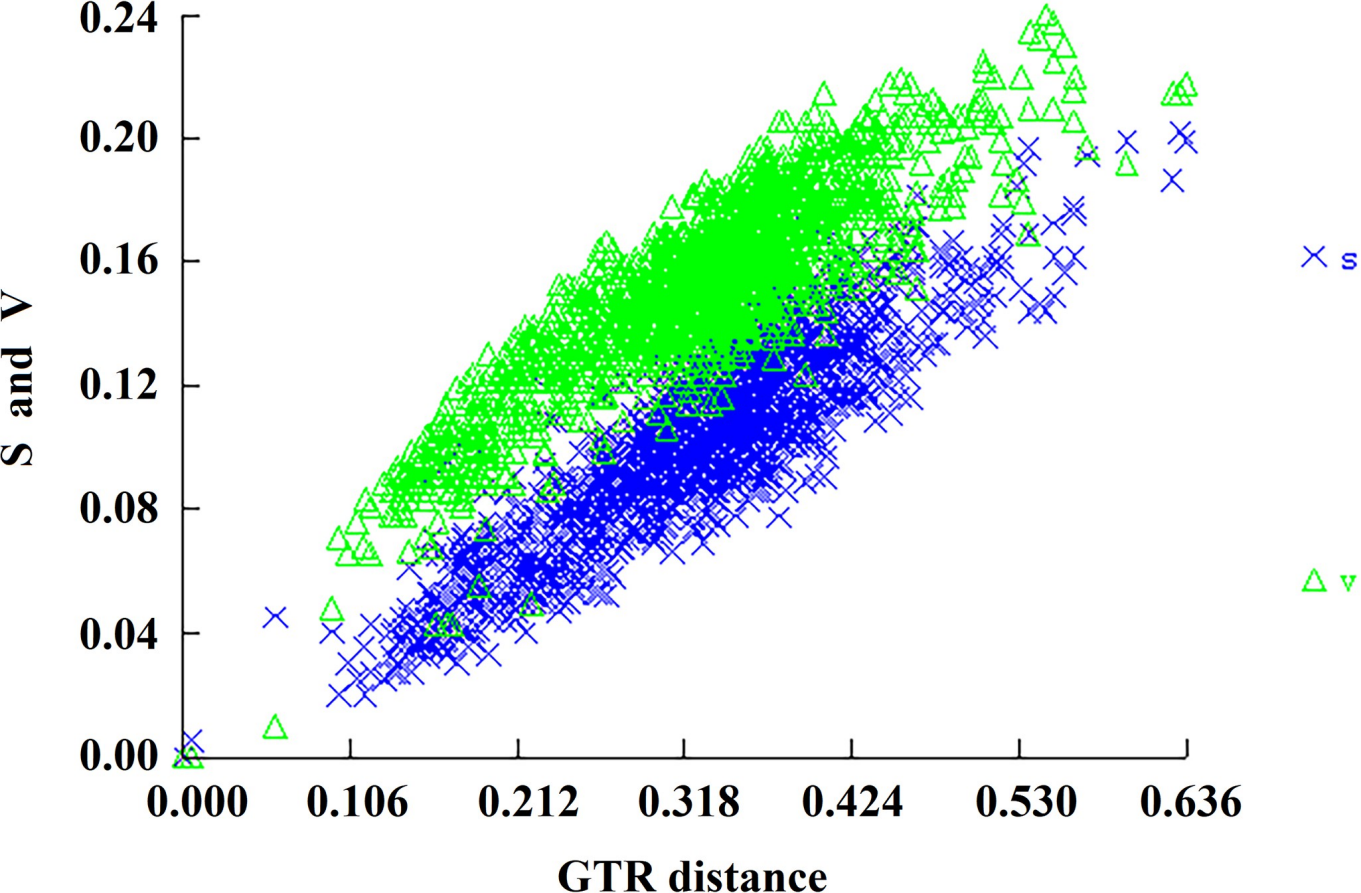
Saturation analysis of base substitutions in species of the family Laelapidae.

The *cox1* genes of 58 species in 19 genera of the family Laelapidae ranged from 404 to 1569 bp in length and from 60.6 to 74.6% in AT content (Table 1). The sequence lengths of the five species determined in this study ranged from 404 to 438 bp; the AT content ranged from 60.6 to 73.8%. The sequences of these five species were compared and trimmed with those of other Laelapidae family species in GenBank to provide an equal sequence length. In the 401 bp alignment of the *cox1* gene sequence, there were 135 conserved sites, 266 variable sites, and 244 parsimony- informative sites. The ratio of non-synonymous mutation rate (Ka) and synonymous mutation rate (Ks) of *cox1* gene sequences of the family Laelapidae was 3.95. Base substitution saturation analysis (Fig 1) showed that the family Laelapidae species showed a linear increase in both base conversion and reversal with increasing genetic distance without a plateau, indicating that the *cox1* gene of the family Laelapidae species is rich in genetic variation and that this gene has great evolutionary potential in species evolution and can be used for phylogenetic analysis.

### Phylogenetic analysis

**Fig 2:**
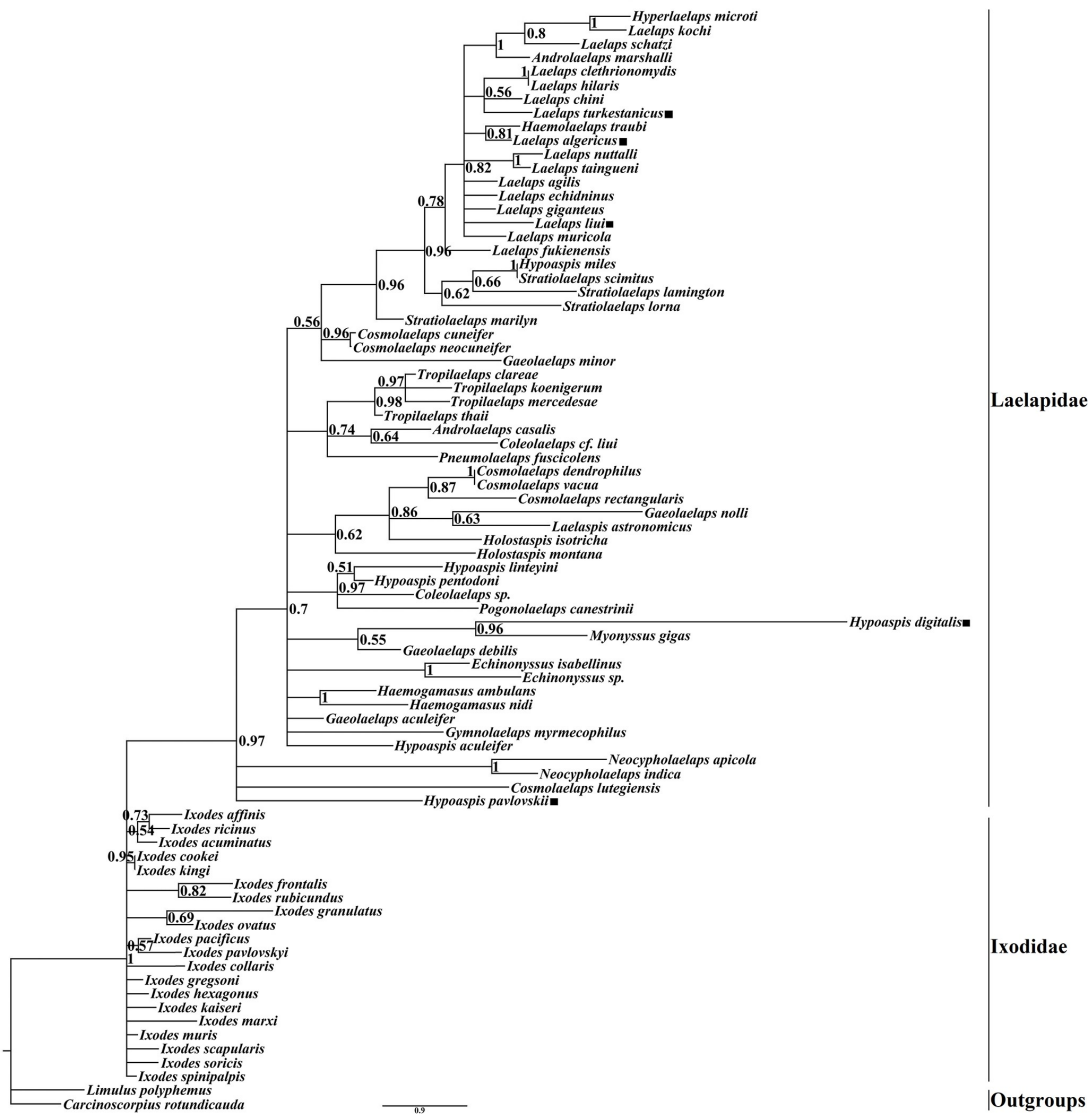
Bayesian phylogenetic tree constructed based on the *cox1* gene sequence. The number on each node indicates the posterior probability (PP) and ■ indicates the species in this study.

**Fig 3:**
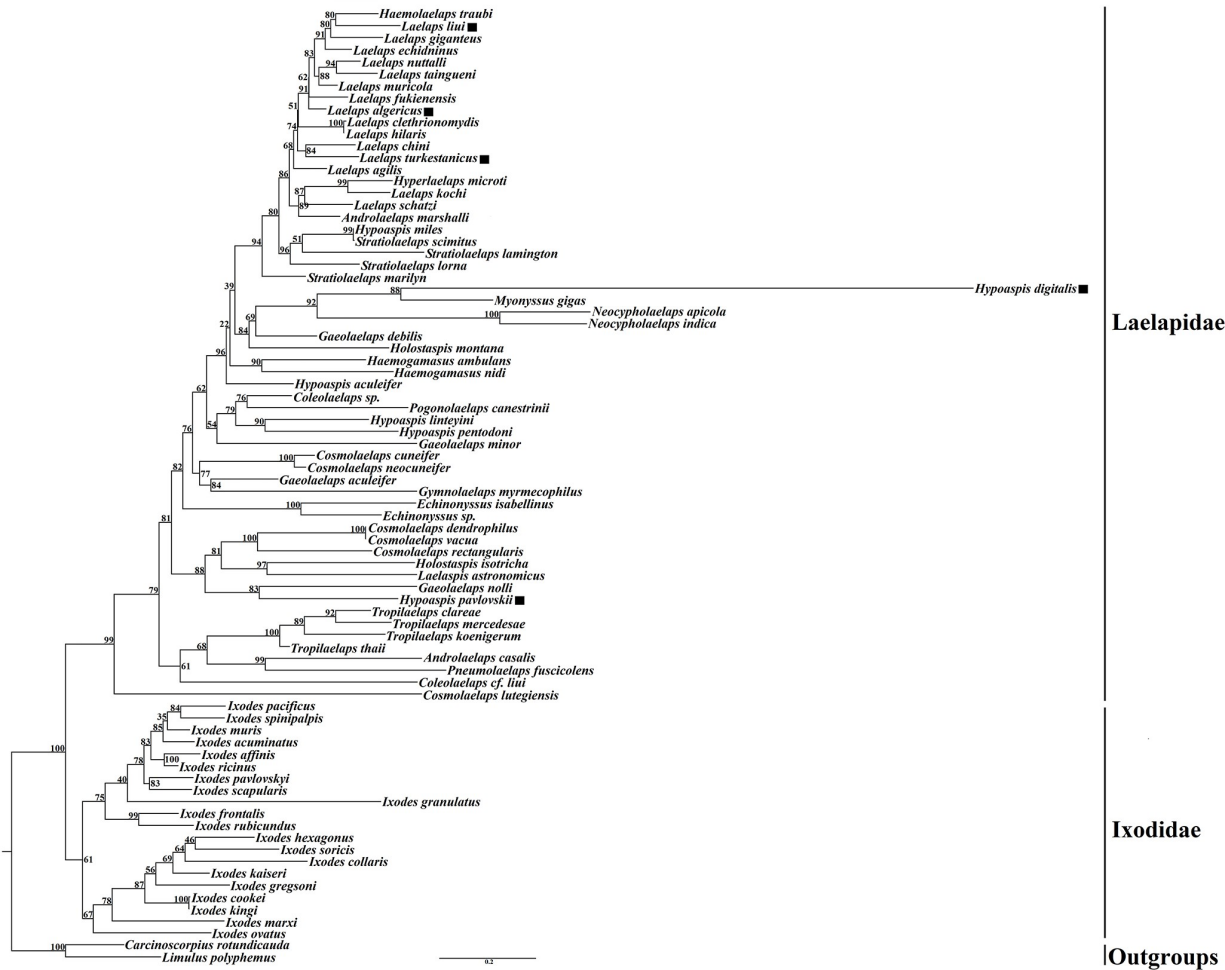
Maximum-likelihood tree constructed based on the *cox1* gene sequence. The number on each node indicates the bootstrap probability (BP) and ■ indicates the species in this study.

Phylogenetic trees of the family Laelapidae were constructed based on the mitochondrial *cox1* gene using *Limulus polyphemus* and *Carcinoscorpius rotundicauda* as outgroups by the BI and ML methods (Figs 2 and 3). The phylogenetic trees constructed by both methods differed slightly in topology and support, but both supported the monophyly of the family Laelapidae (BP = 99, PP = 0.97). For the five species in this study, both ML and BI trees showed that three species (*Laelaps liui*, *Laelaps turkestanicus*, and *Laelaps algericus*) clustered with species of the genus Laelaps, while the other two species (*Hypoaspis pavlovskii* (*Hypoaspis pavloskii* as synonym of *Androlaelaps pavlovskii*) and *Hypoaspis digitalis*) were not clustered with species of the genus *Hypoaspis*. Species within five genera (genera *Laelaps*, *Haemogamasus*, *Tropilaelaps*, *Echinonyssus*, and *Neocypholaelaps*) always cluster together preferentially with high support; seven genera (genera *Stratiolaelaps*, *Gaeolaelaps*, *Coleolaelaps*, *Cosmolaelaps*, *Holostaspis*, *Hypoaspis*, and *Androlaelaps*) have unclear phylogenetic relationships and all form sister branches with high nodal support with species of other genera. Notably, the BI and ML trees show that species of the genera *Haemolaelaps*, *Hyperlaelaps*, and *Androlaelaps marshalli* all cluster together with species of the genus *Laelaps* to form sister branches.

#### Divergence time estimation

**Fig 4:**
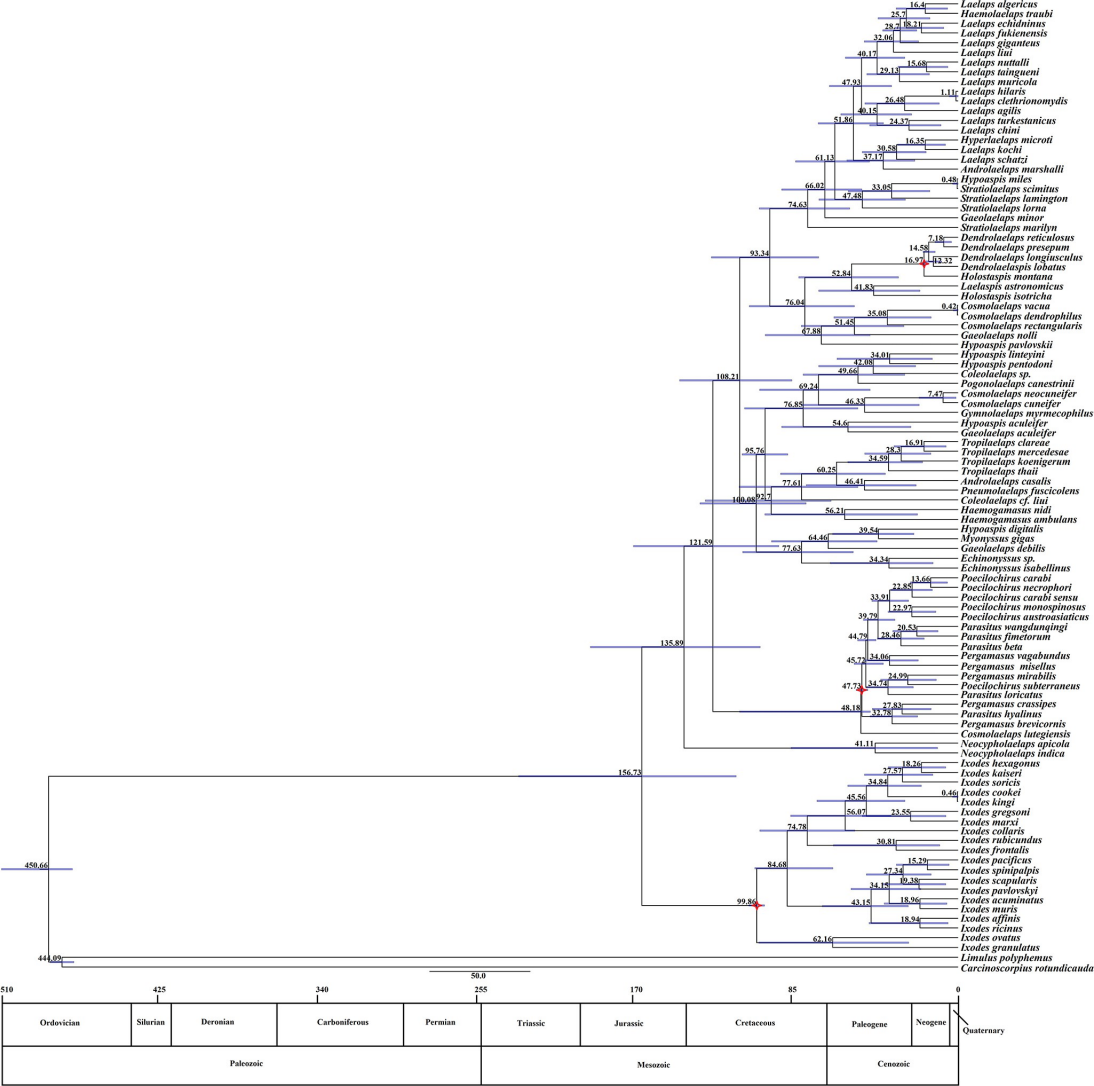
Estimation of the divergence time of the family Laelapidae. (Blue horizontal bars at nodes represent 95% confidence intervals; red star indicates each node calibrated using fossil constraints; numbers on the branches represent the years of divergence, unit: million years).

The origin and divergence times of the major taxa of the family Laelapidae were inferred by using three fossil evidence as calibration points (Fig 4). From Fig 4, the family Laelapidae diverged from other taxa during the Middle Jurassic (~156.73 Mya). The first diversity event within the family Laelapidae occurred at 135.89Mya shortly after the family diverged, separating the genus *Neocypholaelaps* from the species of other genus. Interclade divergence occurred at 100.08Mya and 93.34 Mya. The divergence events of the species of each genus were mainly concentrated in the Cenozoic Tertiary. The timing of species divergence within the genera *Tropilaelaps*, *Haemogamasus*, *Laelaps*, *Echinonyssus*, *Gaeolaelaps* and *Neocypholaelaps* is relatively clustered. With four genera (*Coleolaelaps*, *Hypoaspis*, *Cosmolaelaps*, and *Stratiolaelaps*) had a large divergence time span; among them, species of the genus *Stratiolaelaps* had the largest divergence time span (0.48–74.63Mya), followed by species of the genus *Hypoaspis* (0.48–67.88 Mya). The genera *Myonyssus* and *Gymnolaelaps* diverged from the common ancestor of some species of the genera *Hypoaspis* and *Cosmolaelaps* in 39.54 Mya and 46.33 Mya, respectively. The wide variety of the genus *Laelaps* radiates away mainly around 15–30 Mya. The genera *Haemolaelaps* and *Hyperlaelaps* diverged from species of the genus *Laelaps* at 16.44 Mya and 16.35 Mya, respectively.

#### Infinite-sites plot

**Fig 5:**
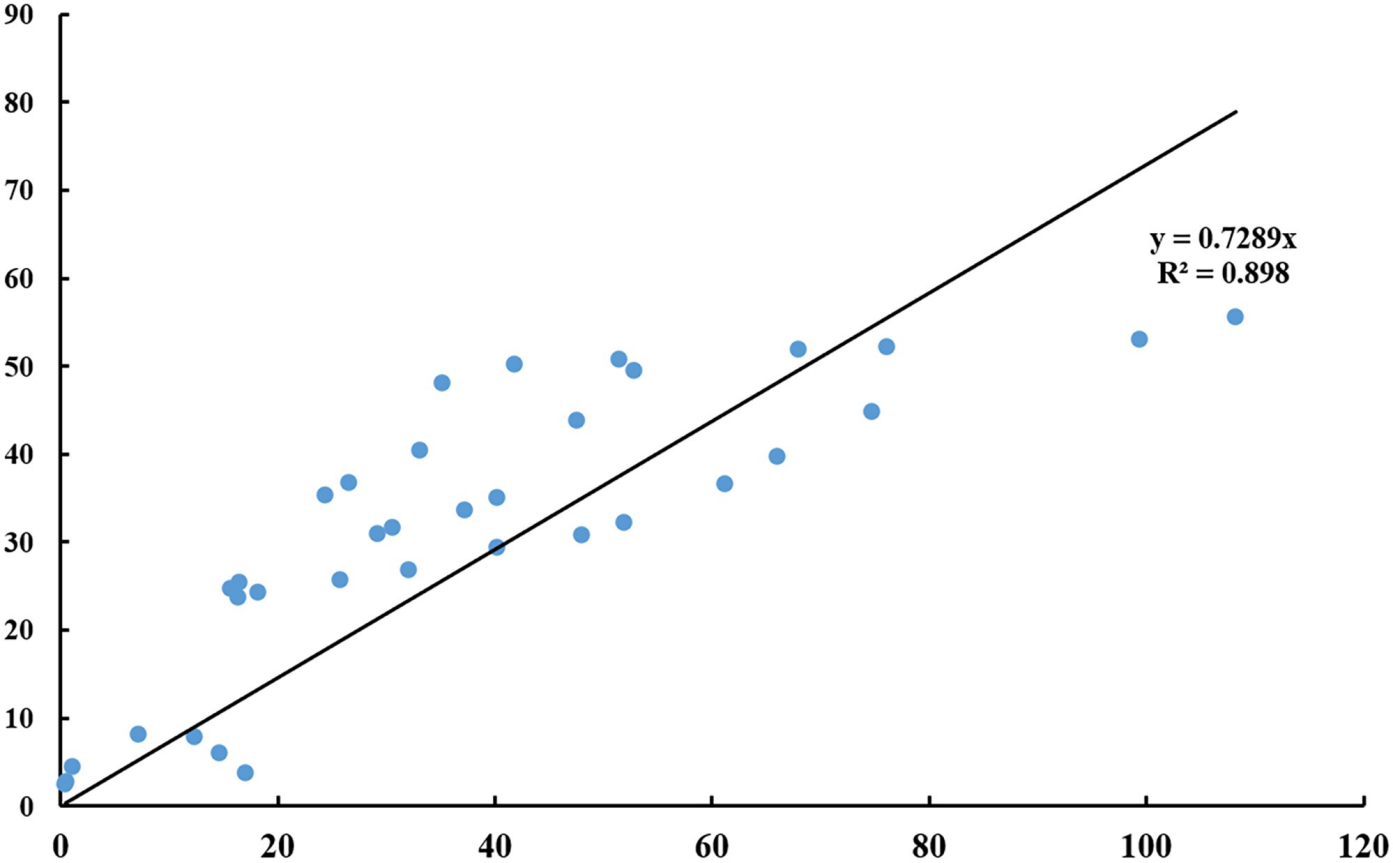
Infinite-sites plot. The plot shows the estimated posterior mean times versus the estimated posterior confidence interval (CI) widths.

Yang and Rannala (2006) proposed an "infinite-sites" theory prediction that compares the posterior interval width to the posterior mean [48]. As the amount of sequence data approaches infinity, the time posterior averages and 95% CIs of different nodes will fall in a straight line. We plotted the width of the posterior 95% CIs against the posterior mean of the node ages to assess whether the amount of sequence data was approaching saturation or whether additional sequence data might improve the precision of the estimates. Fig 5 shows an infinite-sites plot of the data from this study, with a linear relationship between the posterior age mean estimates and the confidence interval in our analysis. The gradient is generally used as a measure of fossil precision, and a gradient of 0.7289 in Fig 5 implies that every 1 Myr of divergence time adds 0.7289 Myr of uncertainty to the posterior estimate (or 0.7289 Myr in the 95% posterior CI interval). The r^2^ = 0.898 indicates that the sequence data are quite limited and far from saturation; adding new genes or species would improve the accuracy of the estimates.

#### Historical biogeography

According to RASP, the best-fitting model for reconstructing the possible biogeographic history of the family Laelapidae is Dispersal-Extinction-Cladogenesis (DEC). The results of the ancestral region reconstruction of the family Laelapidae are shown in Fig 5. DEC analysis postulated that Asia (node 118) is the probable ancestral region of the family Laelapidae (100%). The existing distribution pattern of the species of the family Laelapidae was formed after a complex biogeographic event (26 dispersal events, 16 metamorphic events, and 2 extinction events). Based on the results of the ancestral region reconstruction, it can be found that dispersal and migration events played an important role in the evolution of the family Laelapidae species. Firstly, in evolutionary branch I (node 115), a dispersal event occurred, with some species spreading from Asia to Europe (node 113) and from Europe back to Asia (node 112). In evolutionary branch II (node 108), some species diversified in situ in Asia. Most of the dispersal events occurred in evolutionary branch III (node 102), where firstly, some species diversified in situ in North America and Asia (nodes 101 and 99), and also some species dispersed from Europe to Oceania (node 94) and Asia (node 92). Second, three dispersal events occurred at node 89 (Europe to North America (node 88) and Asia to Europe twice (nodes 86 and 84)); Lastly, a lineage first diversifies in situ in Oceania and then disperses to Europe (node 79), followed by dispersal from Europe to Asia (nodes 73 and 74), then starts in Asia (node 71), disperses through Africa (nodes 68 and 69) to North America and Europe (node 67), and finally follows the North America-Asia-Africa pathway back to Asia (node 61).

## Discussion

It is generally accepted that synonymous mutations are not subject to natural selection, while nonsynonymous mutations are subject to natural selection. When Ka/Ks = 1, there is neutral selection; when Ka/Ks < 1, there is experienced negative selection or purifying selection effect; and when Ka/Ks > 1, there is experienced positive selection or directed selection effect [49–51]. Organisms adapt to their natural environment and survive and reproduce under selective pressures. Positive selection often involves adaptive evolution, resulting in functional innovation [52, 53]. In the present study, we found that the average Ka/Ks ratio of mites in the family Laelapidae was greater than 1 (Ka/Ks = 3.95), and the Ka value was much greater than the Ks value, indicating that species in the family Laelapidae experienced the effect of positive selection, further suggesting that species in the family Laelapidae gradually adapted to such changes by retaining their genetic variation characteristics favorable to adaptation to different geological periods and different hosts and eliminating those unfavorable to evolution during the long-term evolutionary process.

Both the BI and ML trees constructed in this study indicate that the family Laelapidae is a monophyletic group, which is consistent with the findings of Li et al. (2019) [20]. The two phylogenetic trees differed slightly in topology and support, but most nodes had high posterior probabilities (PP) and bootstrap support values (BP). Intergeneric species of five genera (*Laelaps*, *Haemogamasus*, *Tropilaelaps*, *Echinonyssus*, and *Neocypholaelaps*) clustered together preferentially with high nodal support, suggesting that intergeneric variation in these five genera is less than intergeneric variation. According to previous studies, Till (1963) and Furman (1972) grouped species of both the genera *Haemolaelaps* and *Androlaelaps* into the genus *Androlaelaps*, and Berlese (1910) originally created the genus *Haemolaelaps* as a subgenus of the genus *Laelaps*. Zakhvatkin (1948) originally proposed the genus *Hyperlaelaps* as a subgenus of the genus *Laelaps* [14, 54–57]; however, these proposed conclusions have not been shared by most scholars. Therefore, in the current taxonomic studies, the genera *Haemolaelaps*, *Hyperlaelaps*, and *Androlaelaps* are considered as three separate genera of the family Laelapidae, respectively, and they are not studied as subgenera of the genus *Laelaps*. The two phylogenetic trees of this study showed that *Androlaelaps marshalli* of the genus *Androlaelaps*, as well as the species of the genera *Haemolaelaps* and *Hyperlaelaps* all formed sister branches with species of the genus *Laelaps* with high node support (PP>0.80, BP≥80) and did not form a monophyletic branch. Therefore, based on the results of the present study, it is suggested that the genera *Androlaelaps*, *Hyperlaelaps*, and *Haemolaelaps* can be treated as subgenera of the genus *Laelaps* for subsequent related studies. However, the validity of this proposed hypothesis remains to be determined by more extensive and in-depth related studies on other species in these three genera in order to draw more definite conclusions.

The intergeneric relationships of the seven genera (*Stratiolaelaps*, *Gaeolaelaps*, *Coleolaelaps*, *Cosmolaelaps*, *Holostaspis*, *Hypoaspis*, and *Androlaelaps*) are confusing, with some species not clustered with species of the same genus but with species of other genera, forming sister branches. This result is inconsistent with previous taxonomic results observed by morphological phylogeny. The reason for this may be that earlier studies on the classification of species in the family Laelapidae were mainly based on detailed observations of morphological characters and verification of anatomical structures, but because the external morphological characters of some closely related species, complexes, and sister species do not differ much, and because the morphological characters also vary with age, developmental stage, environment, and other factors [58–60], making it difficult to distinguish some Laelapidae family species, resulting in many problems in their species and intergeneric relationships; therefore, inconsistent conclusions are likely to be reached in the traditional morphological classification and phylogenetic studies of the family Laelapidae. It is also possible that the species and numbers used for analysis in this study were small, and the lack of representative species did not reflect the true situation of the phylogenetic tree. With the same analysis method, the number of species covered by the sampling differed significantly, and the phylogenetic trees formed would also differ [61]. In addition, the above situation (i.e., species of different genera closely clustered together) has occurred in the phylogenetic studies on the *Sesarmid crab*, which led to the realignment of related genera and species and the establishment of new genera [62]. Such situations (and clustering of species in different genera) also occur in the family Laelapidae, so that the species of some genera in the family Laelapidae may need to be redefined or new genera may need to be established.

**Fig 6:**
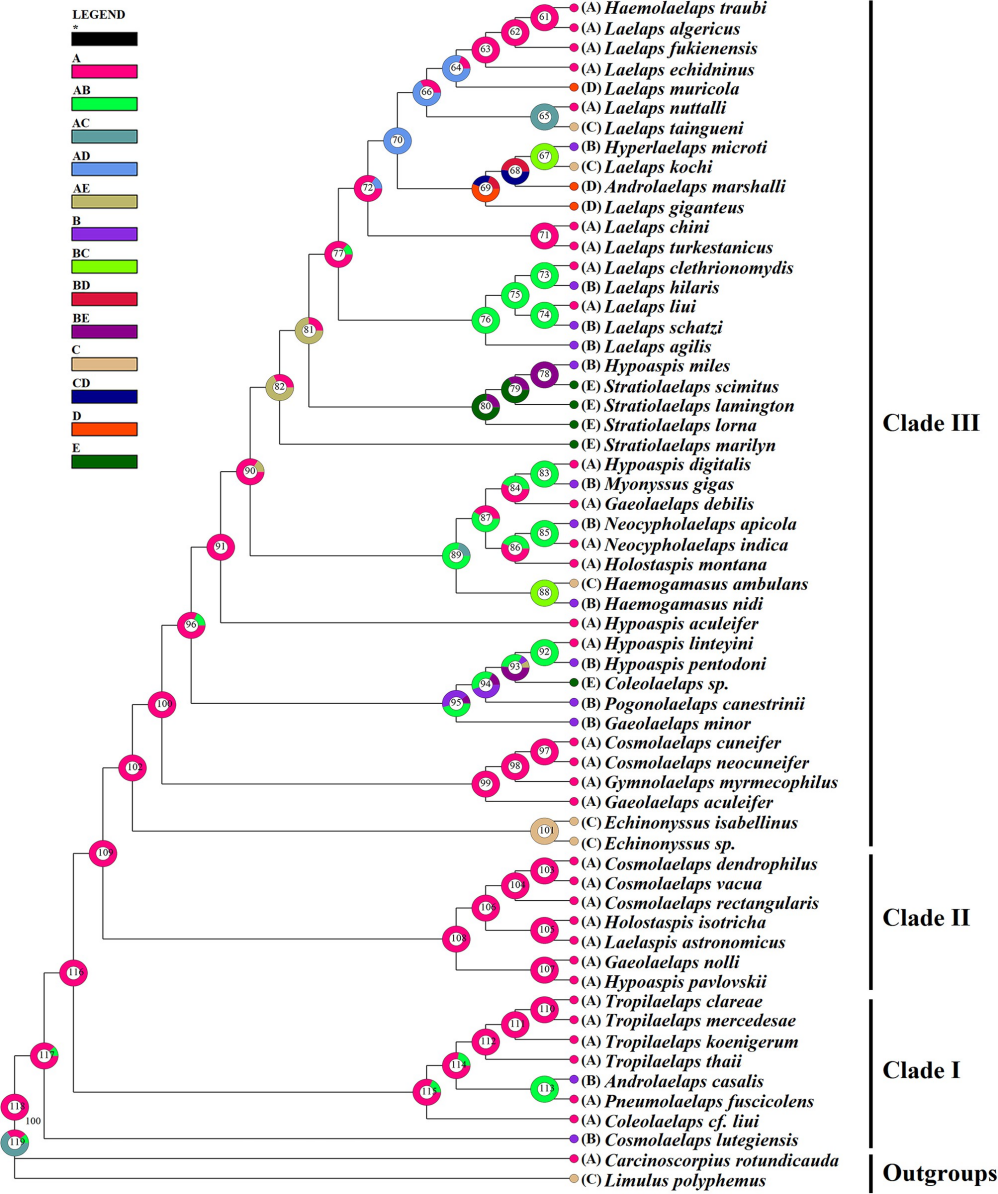
Graphical output of the ancestral distribution of the family Laelapidae obtained using the Dispersal-Extinction-Cladogenesis (DEC) method (exported from RASP). The colors of different nodes represent the range of possible ancestors.

The present study analyzed the divergence time and biogeographic history of species in the Laelapidae family, and the results showed that the family Laelapidae likely diverged from other taxa during the Middle Jurassic (maximum crown age estimated to be about 156.73Mya) (Fig 3), and Asia was inferred to be the most likely ancestral region for the family Laelapidae (Fig 6). Asia is also considered to be a center of origin and diversification of other taxa, including toad, reptiles and birds, etc [63–65]. The first diversity event within the family Laelapidae occurred at 135.89 Mya, splitting the genus *Neocypholaelaps* from the rest of the species in each genus. During the Late Cretaceous (65.5–98.9 Mya), the split between genera was more pronounced, and it has been suggested that the Late Cretaceous fauna entered Asia from Africa and Madagascar via the northward drifting Indian subcontinent, and this event is considered to be a major driver of biodiversity [66]. In addition, the Cretaceous-Paleocene event also occurred during the Late Cretaceous, which represents the end of the Mesozoic and the beginning of the Cenozoic. Four species (*Coleolaelaps cf*. *Liui*, *Hypoaspis pavlovskii*, *Gaeolaelaps minor* *and Stratiolaelaps marilyn*) diverged in Asia, Europe, and Oceania, respectively, suggesting that at least some Laelapidae family species spanned the Cretaceous-Paleogene extinction event (K-Pg). It was not until the Cenozoic Tertiary period that species of various genera of the family Laelapidae began to undergo large-scale diversification events. Studies based on evidence from the fossil record suggest that the Cenozoic Tertiary was a period in mammalian history when reproduction and evolution reached their peak [67]. During the early Cenozoic (55–65 Mya), tectonic collisions between India and Asia formed the Qinghai-Tibet Plateau and the Himalayas, leading to major biogenic exchanges from the former Gondwana continent to Asia. During this period, species of the genera *Gaeolaelaps* and *Stratiolaelaps* underwent a second divergence event in Asia and Oceania, respectively, species of the genus *Haemogamasus* in Europe (*Haemogamasus nidi*) and North America (*Haemogamasus ambulans*) ended their divergence.

During the Eocene period (33.7–55.0 Mya), a large number of genera of the family Laelapidae (genera *Pogonolaelaps*, *Gymnolaelaps*, *Pneumolaelaps*, *Laelaspis*, *Myonyssus*, *Holostaspis* and *Tropilaelaps*) began to emerge in Asia and Europe; the genera *Coleolaelaps* and *Echinonyssus* ended their divergence in Oceania and North America, respectively; the genera *Gaeolaelaps* and *Neocypholaelaps* ended their divergence in Asia and Europe, respectively; the spread of species of the genus *Androlaelaps* from Europe to Africa ended; the genus *Hypoaspis* spread from Asia to Africa; species of the genus *Hypoaspis* undergo diversification in Asia; species of the genera *Stratiolaelaps* began a second divergence event in Oceania and Asia, and species of the genus *Cosmolaelaps* spread from Europe to Asia. At the same time, mammals began to emerge during the Eocene, due to the hot climate and warm oceanic conditions that created a humid and mild environment conducive to species reproduction and the first spread of most (not all) mammalian taxa from Asia to North America and Europe through the Bering Strait [68]. Hence, it can be assumed that the species of the family Laelapidae underwent a large-scale diversification event during the Eocene period associated with the spread of mammals.

The Eocene-Oligocene transition is considered to be an important phase of the Cenozoic. A significant cooling and drying event occurred on a global scale, leading to mass extinctions. After this dramatic transition, the Oligocene period (23.8–33.7 Mya), with a warmer and wetter climate, was entered. During this period, the genera *Stratiolaelaps* and *Tropilaelaps* as well as some species of the genus *Laelaps* underwent divergence. In the early Miocene (16.4–23.8 Mya), the collision between Arabia and Eurasia closed the shipping lanes connecting the Indian and Atlantic Oceans [69], so much so that species of the family Laelapidae did not spread outward during this time period. Only some species of the genus *Laelaps* underwent diversification events in Asia, while the genera *Haemolaelaps*, *Holostaspis*, and *Tropilaelaps* ended up diverging in Asia. During the late Miocene (5.32–7.12 Mya), central Eurasia was strongly influenced by two major water masses (Mediterranean in the south and Eastern Palatinate in the center), which severely affected the development of aquatic fauna and the migration of terrestrial animals (including humans) [70]. During this period, no species of the family Laelapidae underwent divergence. In the Cenozoic Quaternary (0.01–1.81 Mya), the ice age and interglacial period alternated, accompanied by glacial activity, the humid and hot climate zone narrowed southward, leading to the migration of warm-loving flora and fauna toward the equator, and the migration and exchange of plants and animals occurred. During the interglacial period, the climate rebounded, the climatic zone and biota moved towards the poles again, and the biota reappeared vibrant [71]. At this time, six species (*Laelaps hilaris*, *L*. *clethrionomydis*, *Stratiolaelaps sciiics*, *Hypoaspis miles*, *Cosmolaelaps vacua* and *C*. *endendrophilus*) diverge during the alternating ice and interglacial periods.

In summary, species divergence and dispersal within the family Laelapidae could only have occurred during the warm period from the Late Cretaceous to the Late Neogene. The intra-generic species divergence of the genera *Hypoaspis* and *Stratiolaelaps* spanned a large period of time, and these two genera experienced all the events that occurred during the Late Cretaceous to Cenozoic Quaternary (e.g. plate collision, continental drift, climate zonation and temperature changes, and uplift of the Tibetan Plateau etc.), which may be led to some influence on the evolution and development of life in these two genera. Due to limitations in the acquisition of species specimens, the genera *Pogonolaelaps*, *Gymnolaelaps*, *Pneumolaelaps*, *Laelaspis*, *Hyperlaelaps*, and *Haemolaelaps* contain only one species, and this study can only prove that they diverged during the Eocene, but not whether they first diverged. Most genera and species of the family Laelapidae diverged and spread during the Cenozoic Tertiary period. This is because one of the geological events that marked the Cenozoic Tertiary period, the uplift of the Qinghai-Tibetan Plateau, accompanied by several uplift events, occurred during this period. The uplift events cut off genetic exchange between populations through geographic isolation, thus facilitating genetic divergence between populations and eventually leading to the formation of new species [72]. In addition, the climate during the Cenozoic Tertiary period was warmer, wetter, and less variable than during the Mesozoic period, and the warm and humid climate not only promoted the divergence and biodiversity of mites, but also facilitated the survival and reproduction of mites. The constant interaction between organisms and the geographic environment in which they live creates the conditions for the existing biodiversity [73]. Therefore, the divergence and dispersal of species in the family Laelapidae is most likely a joint response to the continued northward drift of the Indian plate closer to Asia during the Late Cretaceous period as it separated from the Gondwana paleo-continent and to the geological activity of the Qinghai-Tibet Plateau during the Cenozoic Tertiary. However, further studies are needed to confirm this.

Since the phylogenetic tree constructed using the *cox1* gene in this study showed that the molecular classification of some species of the Laelapidae family did not fully consistent with the traditional morphological classification results. It might be because of the limited amount of information contained in the *cox1* gene fragment, and the evolutionary tree based on a single or partial gene could not accurately reflect the true phylogenetic relationships among species [74]. Convergence mutation and reverse mutation could mask the true phylogenetic relationships due to random errors [75]. Meanwhile, species of the family Laelapidae are mainly parasitic on the body surfaces of rodents, which further increases the difficulty of collecting specimens. The small size of the family Laelapidae makes it easy to miss the collection of specimens. Thus, in future studies, it is proposed that more comprehensive studies on the morphological and molecular aspects of the family Laelapidae species are needed, and that phylogenetic, divergence time estimates, and biogeographic historical analyses based on complete mitochondrial genome sequences can more accurately reflect the origin and evolution of different genus and species in the family Laelapidae.

## Conclusion

In this study, we analyzed the phylogeny relationships, divergence time, and biogeographic historical of species of the family Laelapidae based on mitochondrial barcoding region. It was found that the family Laelapidae is a monophyletic group, and the species of some genera may need to be redefined or new genera may need to be established. The divergence time and biogeographic historical analyses indicated that the maximum crown age of the family Laelapidae was estimated to be 156.73 Mya, and Asia was inferred as the most likely ancestral regions of the family Laelapidae. It is necessary to increase the species, number of taxonomic orders, and more barcoding region sequences within the family Laelapidae in future studies to further understand the origin, evolution, and divergence of species in the family Laelapidae.
